# Effects of short-term dietary restriction on plasma metabolites and the subcutaneous fat area according to metabolic status in obese individuals: a case–control study

**DOI:** 10.1186/s13098-021-00679-8

**Published:** 2021-06-07

**Authors:** Hye Yoon Jang, Youngmin Han, Hye Jin Yoo, Jong Ho Lee, Minjoo Kim

**Affiliations:** 1 Department of Science for Aging, Graduate School of Yonsei University, Seoul, 03722 Korea; 2 National Leading Research Laboratory of Clinical Nutrigenetics/Nutrigenomics, Department of Food and Nutrition, College of Human Ecology, Yonsei University, Seoul, 03722 Korea; 3 Research Center for Silver Science, Institute of Symbiotic Life-TECH, Yonsei University, Seoul, 03722 Korea; 4 Department of Food and Nutrition, College of Life Science and Nano Technology, Hannam University, Daejeon, 34054 Korea

**Keywords:** Metabolically unhealthy obesity, Metabolically healthy obesity, Dietary restriction, Metabolomics, Leptin, Subcutaneous fat area

## Abstract

**Background:**

Research elucidating the metabolic mechanisms that differentiate subtypes of obesity has been increasing. We aimed to investigate the effects of a 12-week dietary intervention on the metabolomic profiles of obese subjects.

**Methods:**

Subjects followed a 12-week dietary restriction protocol consisting of a 300 kcal/day reduction in their usual caloric intake. Twenty-nine obese subjects were included and divided into two groups: the metabolic status maintenance group (*n* = 17, controls) and the metabolic status improvement group (*n* = 12, tests). We analyzed the somatometric and biochemical parameters and performed ultra-performance liquid chromatography-mass spectrometry analysis of the plasma metabolites.

**Results:**

At 12 weeks, the fat percentage, whole fat area (WFA), subcutaneous fat area (SFA) at the L1 vertebra, and the levels of triglycerides, gamma-glutamyltransferase (gamma-GT), and leptin were markedly decreased in the metabolic status improvement group, while the level of high-density lipoprotein cholesterol increased compared with that in the metabolic status maintenance group. Metabolomic profiling at 12 weeks showed substantial differences in 4-aminobutyraldehyde (*p* = 0.005) and 4’-apo-β-carotenal (*p* = 0.024) between the two groups. Furthermore, an AUC value of 0.89 was obtained for the following seven featured biomarkers: triglycerides, gamma-GT, leptin, fat percentage, WFA, and SFA at the L1 vertebra, and 4-aminobutyraldehyde.

**Conclusions:**

We demonstrated that 4-aminobutyraldehyde and related regional fat distribution parameters were strongly associated with obesity according to metabolic status. Thus, these biomarkers are potentially valuable in confirming the efficacy of short-term interventions and predicting metabolic status in obese individuals.

*Trials registration:* This study was registered at ClinicalTrials.gov under NCT03135132 (registered 1 May 2017—retrospectively registered).

**Supplementary Information:**

The online version contains supplementary material available at 10.1186/s13098-021-00679-8.

## Background

The population of individuals with metabolically unhealthy obesity (MUO) has a higher incidence of cardiovascular disease (CVD), diabetes, and mortality than individuals with metabolically healthy obesity (MHO) [1, 2]; thus, a significant amount of attention has been directed at elucidating the regulatory mechanisms that differentiate the subtypes of obesity. Recently, a broad metabolomics approach was used to investigate differences among individuals with MUO and MHO and healthy lean controls with regard to the metabolic profile of visceral adipose tissue (VAT) [3]. In addition to confirming substantial alterations in oxidative stress and glucose metabolism markers, much of the research revealed that VAT derived from individuals with MUO is characterized by significant dysregulation of several lipid-related metabolic pathways [3, 4]. A recent study reported a plasma panel of important metabolites that can distinguish individuals with MHO from those with MUO; these metabolites include glycolic acid, lysophosphatidylethanolamines, and lysophosphatidylcholines [5]. Additionally, a plasma sphingolipidome profile of the specific metabolic status signature was identified [3]; however, the clinical relevance of the plasma sphingolipidome remains to be elucidated. Although several cross-sectional case–control studies involving obese individuals have been conducted, the effects of dietary interventions on the metabolomics profile of obesity have not been thoroughly studied thus far.

Based on previous studies, regional body fat distribution, regardless of general obesity, may play a critical role in metabolic syndrome (MetS) [6]. However, the effects of the VAT and subcutaneous adipose tissue (SAT) areas on MetS have been debated. As has been widely reported, most studies have demonstrated that the VAT area is associated with an increased risk of incident MetS and MUO [3, 4]. However, studies of SAT and metabolism in obesity are still limited, and SAT has not been identified as a risk factor for metabolic disorder in obese patients in several studies because it may protect against the incidence of individual MetS components [7]. In contrast, Jiala and Devaraj [8] revealed that patients with MetS have significantly higher levels of macrophages, SAT, and several adipokines.

This study was conducted to identify biomarkers that can be used to determine the metabolic status of obese subjects and validate the efficacy of those biomarkers using a prediction model. To achieve our goals, we evaluated the associations of the VFA, the SFA, and adipokines with distinct plasma metabolites in obese subjects stratified according to their metabolic status.

## Methods

### Study subjects

Obese subjects (25 kg/m^2^ ≤ BMI < 30 kg/m^2^) aged 20–60 years were recruited from the Department of Internal Medicine, Yonsei University Severance Hospital, Seoul, Korea. Subjects with a history of CVD, diabetes, thyroid disease, acute or chronic inflammatory disease and those using medications that affected body weight or energy expenditure were excluded. Additionally, subjects with unstable body weight (weight change > 1 kg within three months before screening) were also excluded.

The diagnostic criteria for abnormal metabolism used in the present study were those in the National Cholesterol Education Program Adult Treatment Panel III for Asians as follows [9]: systolic blood pressure (BP) ≥ 130 mmHg or diastolic BP ≥ 85 mmHg, triglycerides ≥ 150 mg/dL, high-density lipoprotein (HDL) cholesterol < 40 mg/dL in males or < 50 mg/dL in females, fasting glucose ≥ 100 mg/dL, and waist circumference ≥ 90 cm in males or ≥ 85 cm in females.

The aim of this study was thoroughly explained, and written informed consent was obtained from all the subjects. The Institutional Review Boards of Yonsei University and the Yonsei University Severance Hospital approved the study protocol, which complied with the principles of the Declaration of Helsinki.

### Study design and intervention

The basic framework of the present study was based on a previous study [10], and we conducted further subanalyses on the data from a prior study (ClinicalTrials.gov; NCT03135132) (Additional file 1: Figure S1). Subjects followed a 12-week dietary restriction protocol consisting of a 300 kcal/day reduction in their usual caloric intake. The participants were recommended to remove one-third of a bowl of rice per meal per day to more easily achieve a 100 kcal deficit per meal because a bowl of rice has 300 kcal according to the food composition tables from the Rural Development Administration (8th Ed., 2011) of Korea. The usual dietary intake was recommended for the control group. Nutrient intake was determined and calculated as a mean value from the 3-day nutritional record using CAN-pro 3.0 (Korean Nutrition Society, Seoul, Korea), which is a commonly used nutrient analysis software in Korea. Additionally, physical activity was assessed using activity patterns, and the total energy expenditure was calculated using the Harris-Benedict Eq. (10). The assigned dietitian checked each participant’s compliance via biweekly phone interviewing.

The present study consisted of a metabolic status maintenance group (n = 17, control group), which was composed of individuals who continued their usual diet, and a metabolic status improvement group (n = 12, test), which was composed of those who restricted their caloric intake; these groups were selected from among those who finished the 12-week program in the previous study [10]. Among the five indicators of abnormal metabolism described above, people who experienced improvements in more than three indicators due to the dietary intervention were grouped into the test group. The control group showed no improvements in the five indicators.

### Somatometric measurements

Body weight, height, waist circumference, hip circumference, systolic BP, and diastolic BP were collected for all subjects [10]. Somatometric data on abdominal fat distribution were measured at the level of the L1 and L4 vertebrae via computed tomography (CT; GE Medical System HiSpeed Advantage® system, Milwaukee, WI, USA), and body composition, including the fat mass, lean body mass, and fat percentage, was measured via dual-energy X-ray absorptiometry (DEXA; Discovery A, Hologic Inc., Bedford, MA, USA).

### Biochemical measurements

Details of the sample collection were described in a previous study [7]. After a fasting period of 12 h, venous blood specimens were collected and stored at − 70 °C until use in further analysis. Levels of the lipid panel, free fatty acids, a glucose-related panel, serum gamma-glutamyltransferase (gamma-GT) assayed via a modified Sanz method, and serum high-sensitivity C-reactive protein (hs-CRP) assayed via a latex-agglutination turbidimetric immunoassay were measured by a Hitachi 7600 autoanalyzer (Hitachi, Tokyo, Japan). Plasma adiponectin concentrations were measured by an enzyme immunoassay [11], and leptin was measured with a Human Leptin ELISA kit (Millipore, Darmstadt, Germany) by SpectraMax190 (Molecular Devices, Shanghai, China).

### Ultra-performance liquid chromatography-mass spectrometry (UPLC-MS) procedure

Nontargeted metabolite screening was conducted to explore the effects of the 12-week dietary intervention on the metabolomic profiles in obese subjects.

For deproteinization, 800 μL of cold acetonitrile (Wako Pure Chemical Industries, Chuo-ku, Osaka, Japan) was added to 150 μL of plasma; this mixture was vortexed for 30 s, followed by 10 min of incubation at 4 °C and centrifugation (10,000 rpm, 5 min, 4 °C). Then, the metabolite-containing supernatant was transferred to a new 1.5 mL microcentrifuge tube, and the solvent was evaporated under nitrogen. Finally, the dried residue was dissolved in 100 μL of cold methanol (J.T. Baker® Chemicals, Avantor Performance Materials, Inc., PA, USA). The quality control (QC) sample was a pool of all plasma samples. The next steps for the prepared QC sample were the same as those described for the plasma samples.

Each sample (5 μL) was injected onto an Acquity UPLC-BEH-C18 column (Waters, Milford, MA, USA) in the Thermo UPLC system (Ultimate 3000 BioRS, Dionex-Thermo Fisher Scientific, Bremen, Germany). In positive mode, 0.1% formic acid in LC–MS-grade water (Fisher Scientific, Fair Lawn, NJ, USA) and 0.1% formic acid in LC–MS-grade methanol (Fisher Scientific) were used for mobile phases A and B, respectively. The flow rate was constant at 0.4 mL/min, but the volumetric concentration of mobile phase B was changed throughout the 22 min: 0% at 0.0–1.0 min, increased from 0 to 100% from 1.0–16.0 min, maintained at 100% from 16.0–20.0 min, and finally decreased to 0% from 20.0–22.0 min for column re-equilibration. Additionally, the prepared QC sample was injected into every 10th sample using a reference standard to monitor the data quality and reproducibility of each multibatch set. MS analysis was performed on a Q-Exactive Plus Orbitrap (Thermo Fisher Scientific, Waltham, MA, USA) with an electrospray ionization (ESI) source in positive ion mode. The MS data were collected in full-scan mode with a scan range of mass-to-charge (*m/z*) of 80–1,000. The MS conditions were set as follows: spray voltage, 3.5 kV; flow rate nitrogen sheath gas, 40 (arbitrary units); auxiliary gas, 10 (arbitrary units); capillary temperature, 320 °C; S-lens RF level, 50; tube-lens voltage, 80 V; and Aux gas heater temperature, 300 °C.

Using the data analysis software SIEVE 2.2 (Thermo Fisher Scientific), raw data were aligned and processed in reference to the QC sample under our parameter settings: *m/z* width, 5 ppm; retention time width, 2.5 min; and *m/z* range, 50–1,000. After this normalization, the candidate metabolites were searched for in the following online databases for identification: Human Metabolome, Lipid MAPS, Kyoto Encyclopedia of Genes and Genomes (KEGG), MassBank, and ChemSpider. Data were Pareto-scaled and then analyzed by orthogonal projection to latent structures-discriminant analysis (OPLS-DA) using SIMCA-P + 15.0 (Umetrics, Inc., Umeå, Sweden). The validity of our models was assessed by the R^2^Y and Q^2^Y parameters and cross-validation-analysis of variance (CV-ANOVA). Plasma metabolites that importantly discriminated between the two groups were selected based on the variable importance in the projection (VIP) values.

### Statistical analyses

All of the statistical analyses were nonparametric and included the Mann–Whitney *U* test and the Wilcoxon signed-rank test, which were performed using IBM SPSS statistics 25.0 (Chicago, IL, USA). Logistic regression analysis was used to calculate the area under the curve (AUC) and 95% confidence interval (CI). A logarithmic transformation was performed for the skewed variables, and significance is reported based on a two-tailed *p*-value < 0.05. For nominal variables, a chi-square test was performed. To correct the multiple comparison problem of the metabolites, a false discovery rate (FDR)-corrected *q*-value was calculated with the R package fdrtool. Pearson’s correlation coefficient was used to analyze the relationships between variables. A heat map was created to visualize the abundances of the identified metabolites and their relationships with the clinical traits. Additionally, receiver operating characteristic (ROC) curves for the created biomarker model were plotted using MetaboAnalyst 4.0 (https://www.metaboanalyst.ca/).

## Results

### Clinical characteristics

A summary of the overall baseline characteristics and differences in the clinical characteristics of all participants is presented in Table 1. At baseline, there were no significant differences between the metabolic status maintenance and metabolic status improvement groups. At 12 weeks, the levels of triglycerides, gamma-GT, and leptin were markedly decreased and the HDL cholesterol level was increased in the metabolic status improvement group compared with those in the metabolic status maintenance group. Additionally, the DEXA and CT measurements, particularly the fat percentage, whole fat area (WFA) at the L1 vertebra, and SFA at the L1 vertebra, showed significant decreases in the metabolic status improvement group compared to the metabolic status maintenance group at 12 weeks. In the within-group comparisons of the metabolic status improvement group, the levels of body weight (*p* = 0.002), BMI (*p* = 0.002), systolic BP (*p* = 0.049), triglycerides (*p* = 0.034), HDL cholesterol (*p* = 0.012), and leptin (*p* = 0.002) and fat-related data, particularly the fat percentage (*p* = 0.002), lean body mass (*p* = 0.002), WFA at the L1 vertebra (*p* = 0.008), SFA at the L1 vertebra (*p* = 0.002), and visceral fat to subcutaneous fat ratio (VSR) at the L1 vertebra (*p* = 0.002), changed significantly after 12 weeks of dietary restriction. However, no significant change was shown in the within-group comparisons in the metabolic status maintenance group.

**Table 1:** Differences in clinical characteristics between the metabolic status maintenance group and metabolic status improvement group after 12 weeks of the intervention

	Metabolic status maintenance group (*n* = 17)		Metabolic status improvement group (*n* = 12)		*p*	*p*^*‡*^
	0 weeks	12 weeks	0 weeks	12 weeks		
Age (yr)	42.3 ± 2.25		40.8 ± 2.90		0.744	
Male/Female *n*, (%)	6 (35.3)/11 (64.7)		10 (83.3)/2 (16.7)			
Weight (kg)	80.5 ± 2.27	79.9 ± 2.18	85.8 ± 4.17	84.2 ± 3.69^**^	0.263	0.263
BMI (kg/m^2^)	27.7 ± 0.37	27.5 ± 0.36	27.8 ± 0.48	27.5 ± 0.36^**^	0.845	0.879
Waist circumference (cm)	96.8 ± 1.31	96.2 ± 1.26	96.6 ± 1.76	95.9 ± 1.45	0.679	0.983
WHR	0.93 ± 0.01	0.93 ± 0.01	0.92 ± 0.01	0.93 ± 0.01	0.811	1.000
Systolic BP	129.4 ± 2.78	127.5 ± 2.97	124.7 ± 4.61	120.8 ± 4.12^*^	0.616	0.195
Diastolic BP	78.4 ± 1.88	79.5 ± 1.96	75.8 ± 2.81	74.5 ± 2.38	0.679	0.152
Triglycerides (mg/dL)^a^	218.1 ± 8.73	245.3 ± 27.0	225.2 ± 32.8	138.6 ± 16.6^*^	0.616	**0.001**
Total cholesterol (mg/dL)^a^	207.5 ± 9.98	208.1 ± 9.56	214.2 ± 11.5	208.6 ± 12.2	1.000	0.811
HDL cholesterol (mg/dL)^a^	43.1 ± 2.68	43.4 ± 2.79	43.8 ± 2.75	52.8 ± 3.53^*^	0.913	**0.014**
LDL cholesterol (mg/dL)^a^	120.9 ± 9.04	115.6 ± 9.33	125.4 ± 10.4	128.1 ± 9.30	0.879	0.527
Free fatty acids (μEq/L)^a^	604.3 ± 65.1	619.9 ± 70.8	525.8 ± 60.0	630.9 ± 150.8	0.499	0.394
Glucose (mg/dL)^a^	91.8 ± 2.76	91.7 ± 3.00	89.7 ± 3.36	89.0 ± 4.24	0.556	0.711
Insulin (μIU/dL)^a^	14.5 ± 1.75	14.9 ± 1.40	14.7 ± 1.99	12.8 ± 1.72	0.913	0.245
HOMA-IR^a^	3.31 ± 0.42	3.39 ± 0.34	3.26 ± 0.44	2.82 ± 0.37	0.913	0.227
C-peptide (ng/mL)^a^	2.36 ± 0.24	2.37 ± 0.22	2.43 ± 0.27	2.10 ± 0.20	0.777	0.444
Gamma-GT (U/L)^a^	31.9 ± 3.05	32.2 ± 4.12	31.8 ± 11.9	20.3 ± 6.26	0.128	**0.048**
Hs-CRP (mg/L)^a^	1.25 ± 0.26	1.43 ± 0.29	1.22 ± 0.33	1.12 ± 0.33	0.616	0.419
Adiponectin (ng/mL)^a^	3.55 ± 0.35	3.56 ± 0.32	4.05 ± 0.69	3.51 ± 0.56	0.845	0.616
Leptin (ng/mL)^a^	16.2 ± 2.86	16.9 ± 2.64	16.6 ± 1.61	8.72 ± 1.02^**^	0.744	**0.012**
Measurements from DEXA and CT						
Fat percentage (%)^a^	29.3 ± 1.43	29.2 ± 1.23	29.1 ± 1.72	23.4 ± 1.71^**^	0.879	**0.018**
Fat mass (g)^a^	22,162.6 ± 968.8	22,065.4 ± 850.6	20,796.4 ± 1649.4	19,810.4 ± 1449.4	0.471	0.211
Lean body mass (g)^a^	51,905.2 ± 2385.7	51,907.2 ± 2170.8	53,402.5 ± 3365.7	57,030.1 ± 3287.7^**^	0.444	0.097
L1 vertebra						
Whole fat area (cm^2^)^a^	27,334.9 ± 1007.3	27,680.2 ± 1130.6	26,398.8 ± 1727.1	22,986.0 ± 1521.6^**^	0.845	**0.027**
Visceral fat area (cm^2^)^a^	12,292.0 ± 965.0	12,603.7 ± 975.2	12,153.4 ± 1043.4	12,221.4 ± 1046.7	0.913	0.913
Subcutaneous fat area (cm^2^)^a^	15,042.9 ± 1151.0	15,076.5 ± 1081.3	14,245.4 ± 1332.9	10,764.6 ± 1199.0^**^	0.777	**0.021**
VSR^a^	0.94 ± 0.13	0.93 ± 0.12	0.93 ± 0.11	1.31 ± 0.19^**^	0.744	0.128
L4 vertebra						
Whole fat area (cm^2^)^a^	32,172.2 ± 1023.8	31,634.2 ± 1135.9	29,139.8 ± 1677.8	28,609.4 ± 1594.9	0.195	0.325
Visceral fat area (cm^2^)^a^	10,174.7 ± 564.0	10,114.0 ± 601.3	9735.7 ± 877.5	9504.6 ± 836.5	0.586	0.394
Subcutaneous fat area (cm^2^)^a^	21,997.5 ± 984.7	21,520.2 ± 1153.2	19,404.1 ± 1915.2	19,104.8 ± 1846.9	0.263	0.245
VSR^a^	0.48 ± 0.04	0.50 ± 0.05	0.59 ± 0.09	0.58 ± 0.09	0.616	0.616

Bold values indicate statistically significant results

Mean ± S.E

*VSR* visceral fat to subcutaneous fat ratio

^a^Tested following logarithmic transformation

*p*-values derived from the Mann–Whitney *U* test at 0 weeks

*p*^*‡*^-values derived from the Mann–Whitney *U* test at 12 weeks

^*^p-values derived from Wilcoxon signed-rank test within the group

### Evaluation of estimated energy and nutrient intake

The estimated total caloric intakes (TCIs) at baseline were 2150.7 ± 74.8 kcal/d and 2153.4 ± 85.8 kcal/d in the metabolic status maintenance group and metabolic status improvement group (*p* = 0.586), respectively (Additional file 2: Table S1). At 12 weeks, a significant decrease in the percentage of the TCI from carbohydrates (*p* < 0.001) and significant increases in the percentages of the TCI from protein and fat (*p* < 0.001; *p* = 0.016) were observed in the metabolic status improvement group compared to the metabolic status maintenance group. Moreover, the metabolic status improvement group had relatively greater reductions in the estimated TCI (*p* = 0.002) and carbohydrate content (*p* = 0.002) and increases in the protein and fat contents (*p* = 0.002; *p* = 0.010) in the within-group comparisons, although no significant change was shown in the within-group comparisons in the metabolic status maintenance group. Additionally, no significant differences were observed in the total energy expenditure between baseline and the 12-week follow-up in either group (Additional file 2: Table S1).

### Identified plasma metabolites

**Fig. 1:**
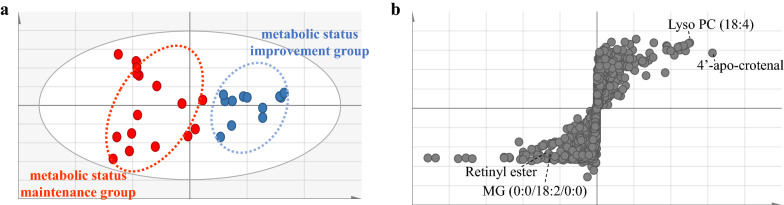
Comparison of plasma metabolites between the metabolic status maintenance group and the metabolic status improvement group at 12 weeks. **A** Score plots from OPLS-DA models classifying follow-up profiles in positive ion mode (*Q*^*2*^ = 0.593, *R*^*2*^*Y* = 0.796). **B** S-plots for covariance (*p*) and reliability correlation [*p(corr)*] from OPLS-DA models in positive ion mode

The results of OPLS-DA in the positive mode for the two groups at 12 weeks are shown in Fig. 1. Following the 12-week intervention, the plasma metabolomes of the two groups markedly differed (*Q*^*2*^ = 0.593, *R*^*2*^*Y* = 0.796). A total of 20 mass features were responsible for the apparent variation in the metabolic fingerprints following the 12-week dietary restriction protocol. Details of individual mass features in which the identified metabolites differed between the two groups at 12 weeks are presented in Table 2. We detected a total of 1,703 features, and nine metabolites (excluding 11 drug compounds and those with a high missing rate of detection) of known identity were identified after the 12-week intervention in both groups. These nine metabolites were selected based on the importance of each metabolite in the projection (VIP > 1.0), and two metabolites achieved statistical significance (*p* < 0.05). 4-Aminobutyraldehyde and 4’-apo-β-carotenal significantly decreased (*p* = 0.005) and increased (*p* = 0.024), respectively, in the metabolic status improvement group (Table 2). Among the features that were not identified, 184 features were those with a VIP > 1.0; those with a high missing detection rate were excluded (Additional file 2 Table S2).

**Table 2:** Untargeted metabolomic profiling in the metabolic status maintenance group and metabolic status improvement group at 12 weeks

Metabolite	Formula	*m/z*	Metabolic status maintenance group (*n* = 17)	Metabolic status improvement group (*n* = 12)	VIP	*p*	*q*
3-Methoxybenzenepropanoic acid	C_10_H_12_O_3_	181.087	48,483,001 ± 2,436,817	47,524,456 ± 2,472,852	1.090	0.777	0.773
3-Oxodecanoic acid	C_10_H_18_O_3_	187.133	17,193,428 ± 5,245,147	18,066 ± 6192	1.123	0.053	0.148
4-Aminobutyraldehyde	C_4_H_9_NO	88.076	731,124,345 ± 52,880,560	521,581,495 ± 28,774,143	1.576	**0.005**	0.079
4'-Apo-β-carotenal	C_35_H_46_O	483.366	178,245,850 ± 29,268,414	281,940,254 ± 20,697,133	8.915	**0.024**	0.115
Creatine	C_4_H_9_N_3_O_2_	132.066	73,998,544 ± 3,083,621	69,558,095 ± 4,435,850	1.867	0.419	0.402
LysoPC (18:4)	C_26_H_46_NO_7_P	516.308	5,405,106,623 ± 430,811,273	6,055,631,630 ± 237,704,258	4.383	0.711	0.536
MG (0:0/18:2/0:0)	C_21_H_38_O_4_	355.367	30,656,117,810 ± 1,869,304,399	29,781,442,820 ± 2,257,208,392	2.244	0.616	0.499
N-Arachidonoyl tyrosine	C_29_H_41_NO_4_	468.311	10,791,398,770 ± 615,655,355	10,063,129,180 ± 816,638,352	1.202	0.303	0.332
Retinyl ester	C_20_H_30_O_2_	302.245	4,362,841,825 ± 1,329,757,698	426,188 ± 132,349	2.324	0.195	0.266

Bold values indicate statistically significant results

Mean ± SE. Mann–Whitney *U* test was used to calculate *p*-values. *q*-values are the false discovery rate (FDR)-adjusted *p*-values

*VIP* variable important in the projection

### Evaluation of biomarkers and their associations with clinical traits

**Fig. 2:**
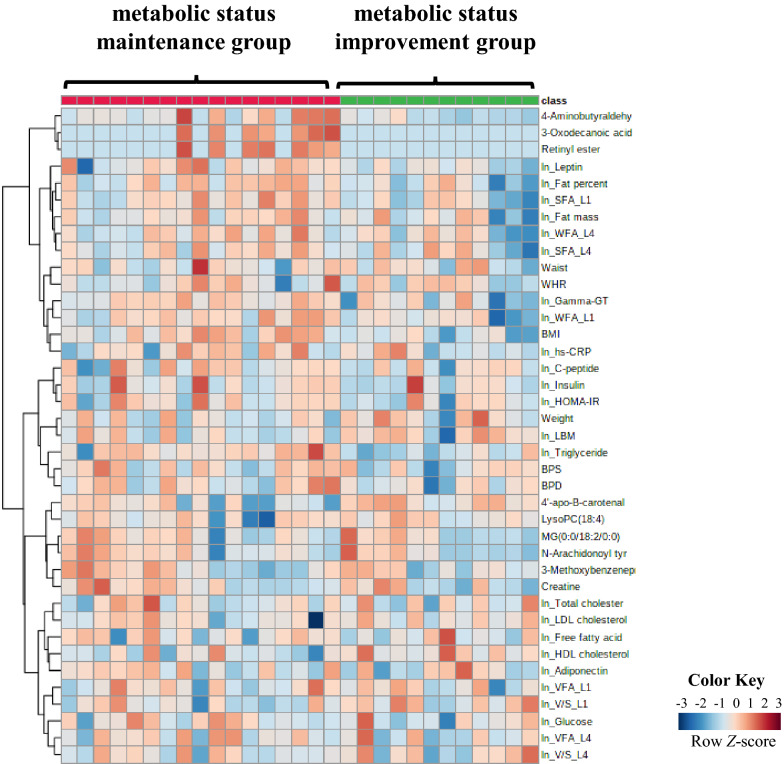
A heat map visualizing the somatometric, biochemical, and metabolomic data. Each row represents a feature measured at 12 weeks, and each column represents a sample. All of the features of the two groups are projected on the heat map. The row *Z*-score or scaled expression value of each feature is plotted in the red-blue color scale. Red indicates high abundance, and blue indicates low abundance. The distance was measured using *Euclidean*, and the clustering algorithm was performed using *Ward*’s method

The relative distributions of the nine differential metabolites in the metabolic status maintenance group and metabolic status improvement group were represented as a set of *Z*-scores. These nine differential metabolites identified in the samples were normalized to the median among the samples. To visualize the metabolic variations, the *Z*-scores of the differential metabolites, somatometrics, and log-transformed biochemical data from the two groups were used to generate a heat map (Fig. 2). The heat map indicated higher values for the clinical traits, except for HDL cholesterol, in the metabolic status maintenance group. At 12 weeks, two differential metabolites, namely, 4-aminobutyraldehyde and 4’-apo-β-carotenal, showed greater metabolic alterations between the two groups than the other identified metabolites.

**Fig. 3:**
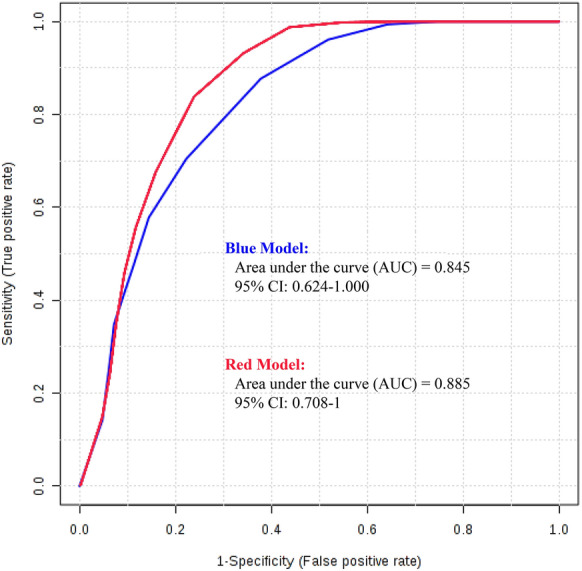
A plot of the ROC curve for the created biomarker model. The blue model consists of triglycerides, HDL cholesterol, gamma-GT, leptin, fat percentage, WFA at the L1 vertebra, SFA at the L1 vertebra, 4-aminobutyraldehyde, and 4’-apo-β-carotenal. The red model consists of triglycerides, gamma-GT, leptin, fat percentage, WFA at the L1 vertebra, SFA at the L1 vertebra, and 4-aminobutyraldehyde

A logistic regression model was constructed to evaluate the predictive ability of each of the ten significant features for improvements in the metabolic state; we used the two metabolites mentioned above, the seven significant clinical traits based on the Mann–Whitney U test at 12 weeks, and BMI, which is a traditional diagnostic criterion for obesity. ROC curves were obtained from a logistic regression model, and the AUC values of each of these ten featured biomarkers were calculated and are shown in Additional file 2: Table S3. An AUC of 0.845 was obtained after excluding two features (BMI and leptin), which indicated a high predictive ability for improvements in the metabolic state (Fig. 3). A higher AUC value of 0.885 was obtained from the curve of the model consisting of triglycerides, gamma-GT, leptin, fat percentage, WFA at the L1 vertebra, SFA at the L1 vertebra, and 4-aminobutyraldehyde.

## Discussion

The present study demonstrated the effects of a 12-week dietary restriction protocol on metabolic status, with profound changes in the levels of triglycerides, HDL cholesterol, gamma-GT, leptin, fat percentage, WFA at the L1 vertebra, and SFA at the L1 vertebra in obese individuals. At 12 weeks, significant differences in the SFA but not the VFA were observed between the metabolic status maintenance and metabolic status improvement groups. Recent studies have proposed the crucial role of SAT dysregulation in MetS [8, 12]. It appears that both inflammation and insulin resistance (IR) play pivotal roles in the significant increase in SAT observed in patients with MetS. Furthermore, there are marked increases in several types of adipokines, including plasma leptin, while the adiponectin concentration decreases [8]. These phenomena were consistent with our findings that the metabolic status maintenance group had higher levels of leptin and a larger area of subcutaneous fat than metabolic status improvement group. However, the adiponectin concentration was not substantially different between the two groups because this adipokine is mainly derived from VAT. The metabolic status maintenance group did not have a significantly higher VFA than the other group; thus, the concentration of adiponectin did not differ between the two groups. This result was similar to a previous study showing that the secretion of adiponectin from VAT decreased with an increase in total body fat [13].

In particular, the WFA, SFA, and VSR of specific regional fat distribution at the L1 vertebra decreased significantly after 12 weeks of dietary restriction in the metabolic status improvement group. As the first vertebra in the lumbar region, the L1 vertebra bears the weight of the upper body and acts as a transition between the thoracic and lumbar vertebrae. The distribution of fat was observed to be primarily concentrated in the lower half of the spine, with a gradual, incremental increase from the upper to lower levels [14]. However, our results did not show significant increases in the WFA at the L4 vertebra compared to the L1 vertebra. Because 12 weeks of dietary restriction is a relatively short duration, it could not effectively influence the WFA despite changes in metabolic status. Because deep SAT (dSAT), rather than superficial SAT (sSAT), is strongly associated with abnormal lipid panels and IR [15–17], it is closely related to the pathophysiology of obesity complications in a manner nearly equivalent to the characteristics of VAT in general [12]. Based on these findings, dSAT might account for most SFA in obese individuals with several cardiometabolic risk factors in the current study. Additionally, we can speculate that dietary restriction markedly affects dSAT in the SFA at the L1 vertebra even though dSAT was not measured. There have been no investigations concerning the effects of dietary restriction on fat loss concerning specific fat distributions; thus, it is difficult to demonstrate their associations. Further study is needed to explain differences in the effects of short-term dietary restriction according to the regional fat distribution.

The decreased leptin level shown in our study could be a key indicator of improved metabolic status accompanied by weight loss [18]. Leptin, which is secreted in proportion to the mass of adipose tissue, is critical for energy balance [19]. Several studies have been aligned with our findings. Minocci et al. [20] demonstrated that SFA is a critical determinant of the leptin concentration regardless of the fat mass in obese patients. Additionally, leptin has been reported to be able to define nutritional status in the elderly population, reflecting metabolic reserves composed of fat, especially peripheral subcutaneous fat [21]. Additionally, leptin has been considered a cardiovascular risk factor in individuals with MHO due to its contribution to the inflammatory process related to obesity-associated cardiometabolic complications [22]. It was confirmed that 12 weeks of nutritional restriction improved the metabolic status of obese subjects, with a direct effect on the concentration of leptin that was directly or indirectly related to the reduction in SFA.

We identified two specific plasma metabolites that are likely to be associated with positive metabolic changes, especially concerning adipose tissue function that may relate to leptin activity. 4-Aminobutyraldehyde and 4’-apo-β-carotenal were markedly altered after 12 weeks of dietary restriction, and profound differences were observed between the two groups.

4-Aminobutyraldehyde, one of the significant biomarkers discovered in our study, is a substrate of aminobutyraldehyde dehydrogenase in arginine, proline, and beta-alanine metabolism (KEGG); 4-aminobutyraldehyde has been shown to cross the blood–brain barrier and be converted rapidly to gamma-aminobutyric acid (GABA) in various regions of the brain [23]. The leptin receptor in GABAergic neurons mediates the significant role of leptin in body weight regulation, suggesting an essential role for GABA release in negotiating leptin action [24, 25]. Collectively, the close crosslink between 4-aminobutyraldehyde and leptin action with GABA has the potential to result in substantial changes in weight, BMI, and fat composition. However, because many other neuropeptides are involved in activating GABAergic neurons, more studies are needed to validate this relationship.

Regarding 4’-apo-β-carotenal, although there is a lack of research concerning this metabolite, the roles of several apo-β-carotenals have been investigated. 14′-Apo-β-carotenal behaves as a weak retinoic acid receptor (RAR) agonist [26, 27], yet its inhibitory effect on adipogenesis has been traced to its ability to suppress peroxisome proliferator-activated receptor (PPAR) γ- and retinoid X receptor (RXR)-mediated responses through RAR-independent mechanisms, possibly following direct physical binding to these receptors [26, 27]. The significantly higher 4’-apo-β-carotenal level in the metabolic status improvement group at 12 weeks than in the metabolic status maintenance group in the current study may have a similar mechanism of action with regard to decreasing fat accumulation. Moreover, β-13-apocarotenone has been shown to inhibit RXRα activity [28], and 10′-apo-β-carotenal has been identified in adipose tissue [29]. In addition to these compounds, it is highly probable that other apocarotenoids are produced in adipose tissue, but their function in adipocyte biology needs further research, including research regarding the currently identified 4’-apo-β-carotenal.

At this time, the exact mechanisms regarding the different effects of short-term dietary restriction in obese individuals have not been fully elucidated. Moreover, the sample size of the present study was small. Therefore, further studies with larger sample sizes are needed to validate our discovery and clarify the exact mechanisms. In addition, a calorie-restricted diet changed the proportions of nutrients consumed by the subjects. To clarify whether the metabolic changes seen in obese subjects was the effect of simple calorie restriction or the effect of changing the nutrient composition, additional studies controlling for more factors should be conducted.

Despite these limitations, we found close associations among BMI, triglycerides, HDL cholesterol, gamma-GT, leptin, fat percentage, WFA, and SFA at L1,4-aminobutyraldehyde and 4’-apo-β-carotenal in obese subjects after 12 weeks of dietary restriction. Additionally, we verified the improved predictive ability with regard to the metabolic status in obese individuals using these significant biomarkers compared to a predictive model composed of traditional obesity indicators only. Notably, 4-aminobutyraldehyde was markedly different between the two groups, and that difference was associated with the leptin levels; furthermore, these two indicators had closer associations with SFA than with VFA. Thus, a metabolite that may be related to leptin and regional fat distribution, *i.e.*, SFA, was strongly correlated with obesity according to metabolic status. To the best of our knowledge, this is the first study to demonstrate the close relationship between metabolic status and SFA at the L1 vertebra rather than the VFA.

## Conclusions

The biomarkers found in this study and their relevance have great potential as the basis of a model confirming the efficacy of short-term interventions and predicting metabolic status in obese individuals.

## Supplementary Information

**Additional file 1: Figure S1.** Flow chart of participants.

**Additional file 2: Table S1. **Comparison of major nutrients’ composition between metabolic status maintenance group and metabolic status improvement group.** Table S2.** Unknown features with VIP > 1.0. ** Table S3**. AUCs for featured biomarkers.

## Data Availability

The datasets generated and/or analyzed during the current study are available from the corresponding author on reasonable request.

## Abbreviations

AUC: Area under the curve
BMI: Body mass index
BP: Blood pressure
CI: Confidence interval
CT: Computed tomography
CVD: Cardiovascular disease
DEXA: Dual-energy X-ray absorptiometry
dSAT: Deep SAT
ESI: Electrospray ionization
FDR: False discovery rate
GABA: Gamma-aminobutyric acid
gamma-GT: Gamma-glutamyltransferase
HDL: High-density lipoprotein
hs-CRP: High-sensitivity C-reactive protein
IR: Insulin resistance
MetS: Metabolic syndrome
MHO: Metabolically healthy obesity
MUO: Metabolically unhealthy obesity
OPLS-DA: Orthogonal projections to latent structures discriminant analysis
PPAR: Peroxisome proliferator-activated receptor
QC: Quality control
ROC: Receiver operating characteristic
RXR: Retinoid X receptor
SAT: Subcutaneous adipose tissue
SFA: Subcutaneous fat area
sSAT: Superficial SAT
TCI: Total caloric intakes
VAT: Visceral adipose tissue
VFA: Visceral fat area
VIP: Variable importance in projection
VSR: Visceral fat to subcutaneous fat ratio
WFA: Whole fat area
