# Supplementary material for: Effects of short-term dietary restriction on plasma metabolites and the subcutaneous fat area according to metabolic status in obese individuals: a case–control study
Source: Diabetol Metab Syndr. 2021 Jun 7;13:62. doi: 10.1186/s13098-021-00679-8 (PMC8186103; doi:10.1186/s13098-021-00679-8)
Supplement: Supplementary file 1 — Additional file 1: Figure S1. Flow chart of participants. [file 13098_2021_679_MOESM1_ESM.pptx]

## Slide 1
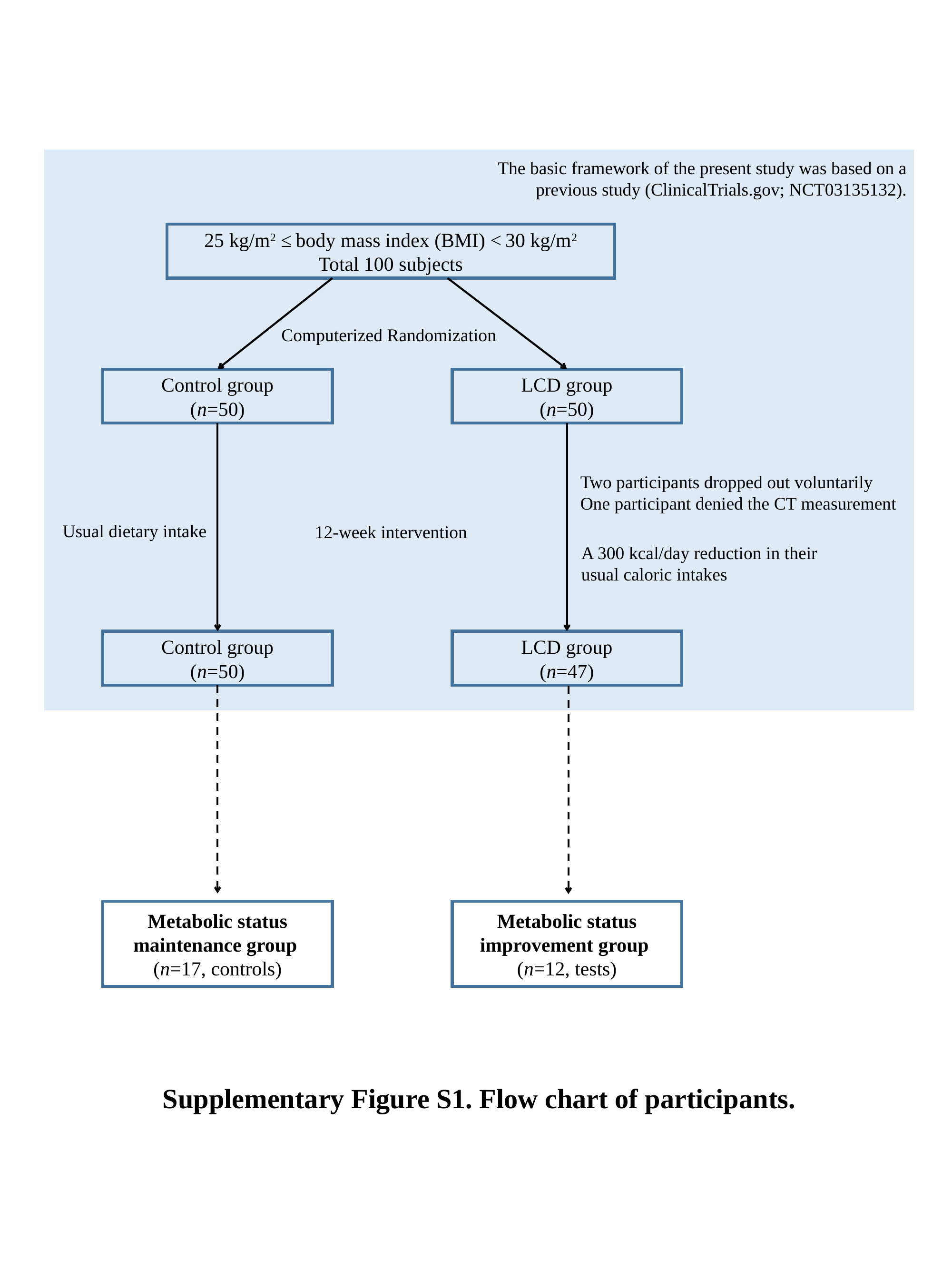

The basic framework of the present study was based on a previous study (ClinicalTrials.gov; NCT03135132).
25 kg/m2 ≤ body mass index (BMI) < 30 kg/m2
Total 100 subjects
Computerized Randomization
Control group
(n=50)
LCD group
(n=50)
Two participants dropped out voluntarily
One participant denied the CT measurement
Usual dietary intake
12-week intervention
A 300 kcal/day reduction in their usual caloric intakes
Control group
(n=50)
LCD group
(n=47)
Metabolic status maintenance group
(n=17, controls)
Metabolic status improvement group
(n=12, tests)
Supplementary Figure S1. Flow chart of participants.
