# Supplementary material for: Effects of short-term dietary restriction on plasma metabolites and the subcutaneous fat area according to metabolic status in obese individuals: a case–control study
Source: Diabetol Metab Syndr. 2021 Jun 7;13:62. doi: 10.1186/s13098-021-00679-8 (PMC8186103; doi:10.1186/s13098-021-00679-8)
Supplement: Supplementary file 2 — Additional file 2: Table S1. Comparison of major nutrients’ composition between metabolic status maintenance group and metabolic status improvement group. Table S2. Unknown features with VIP > 1.0. Table S3. AUCs for featured biomarkers. [file 13098_2021_679_MOESM2_ESM.docx]

**Supplementary Table S1. Comparison of major nutrients’ composition between metabolic status maintenance group and metabolic status improvement group.**

|  | **Metabolic status**  **maintenance group (*n*=17)** | | | | | **Metabolic status**  **improvement group (*n*=12)** | | | | | ***p*** | ***p****^‡^* |
| --- | --- | --- | --- | --- | --- | --- | --- | --- | --- | --- | --- | --- |
|  | **Baseline** | | **Follow-up** | | | **Baseline** | | **Follow-up** | | |  |  |
| Total energy expenditure (kcal/d)*^∮^* | 2191.9 | ±78.7 | 2199.5 | ±80.7 | | 2105.4 | ±97.9 | 2124.8 | ±96.4 | | 0.965 | 1.000 |
| Total calorie intake (kcal/d)*^∮^* | 2150.7 | ±74.8 | 2151.4 | ±73.1 | 2153.4 | | ±85.8 | 1839.9 | ±84.8*^**^* | 0.586 | | 0.024 |
| Carbohydrate (%)*^∮^* | 61.6 | ±0.16 | 61.4 | ±0.23 | 61.5 | | ±0.17 | 59.7 | ±0.22*^**^* | 0.471 | | <0.001 |
| Protein (%)*^∮^* | 15.8 | ±0.10 | 15.9 | ±0.10 | 15.8 | | ±0.12 | 17.0 | ±0.12*^**^* | 0.744 | | <0.001 |
| Fat (%)*^∮^* | 22.6 | ±0.23 | 22.7 | ±0.30 | 22.5 | | ±0.23 | 23.9 | ±0.32*^*^* | 0.777 | | 0.016 |

Mean ± S.E. *^†^*Tested following logarithmic transformation. VSR, visceral fat to subcutaneous fat ratio. *p*-values derived from the Mann-Whitney *U* test at 0 weeks. *p^‡^*-values derived from the Mann-Whitney *U* test at 12 weeks. *^*^p*-values derived from Wilcoxon signed-rank test within the group.

**Supplementary Table S2. Unknown features with VIP > 1.0.**

| **Unknown features** | ***m/z*** | **RT  (min)** | **Metabolic status  maintenance group (n=17)** | | **Metabolic status improvement group (n=12)** | | **VIP** |
| --- | --- | --- | --- | --- | --- | --- | --- |
| u1 | 494.326 | 15.442 | 61600 | ±36828 | 18534 | ±13618 | 8.488 |
| u2 | 498.330 | 18.951 | 1174542637 | ±275897169 | 1546321128 | ±227941911 | 6.900 |
| u3 | 642.606 | 20.947 | 1297820407 | ±302522594 | 1708626264 | ±241469676 | 6.450 |
| u4 | 642.602 | 20.924 | 883458791 | ±212716808 | 1194318298 | ±163639201 | 5.372 |
| u5 | 760.577 | 18.976 | 987500789 | ±205728513 | 1205441356 | ±175945159 | 5.021 |
| u6 | 792.579 | 18.847 | 724086034 | ±183826820 | 987097942 | ±131253893 | 4.921 |
| u7 | 809.588 | 18.746 | 966491919 | ±228394341 | 1235327359 | ±134696710 | 4.771 |
| u8 | 432.404 | 17.911 | 802620403 | ±180012332 | 997620754 | ±101583457 | 4.699 |
| u9 | 526.295 | 15.712 | 867627398 | ±196159500 | 1113978608 | ±117198950 | 4.681 |
| u10 | 788.676 | 20.020 | 970657094 | ±230469581 | 1229389576 | ±146404508 | 4.604 |
| u11 | 519.324 | 16.083 | 810877785 | ±184962321 | 1022292073 | ±128103661 | 4.315 |
| u12 | 518.323 | 16.099 | 518351952 | ±95282565 | 570535451 | ±57649343 | 4.160 |
| u13 | 518.327 | 15.301 | 1233444378 | ±206312021 | 1250049430 | ±146638240 | 4.113 |
| u14 | 520.335 | 8.212 | 1197929854 | ±179542555 | 1200141023 | ±128691208 | 4.062 |
| u15 | 526.376 | 16.669 | 1348446439 | ±204609054 | 1229624917 | ±140145799 | 4.014 |
| u16 | 524.373 | 16.667 | 1355764250 | ±201739073 | 1232050514 | ±137391563 | 4.012 |
| u17 | 451.384 | 16.938 | 1291446823 | ±217181999 | 1061581082 | ±241560723 | 3.931 |
| u18 | 519.328 | 16.080 | 1483346074 | ±236141085 | 1235963514 | ±282686983 | 3.894 |
| u19 | 787.595 | 19.140 | 1178504404 | ±168002756 | 934372055 | ±168357692 | 3.877 |
| u20 | 526.370 | 16.684 | 996579674 | ±168985880 | 701787204 | ±173489437 | 3.851 |
| u21 | 526.379 | 16.667 | 955606667 | ±129520850 | 731699653 | ±146872536 | 3.835 |
| u22 | 809.582 | 18.512 | 282829603 | ±92744912 | 10428377 | ±5123752 | 3.814 |
| u23 | 520.339 | 15.716 | 571993638 | ±188823563 | 626173 | ±431627 | 3.799 |
| u24 | 524.370 | 16.669 | 520810190 | ±173900804 | 4587589 | ±2682899 | 3.792 |
| u25 | 524.361 | 16.154 | 480837298 | ±158535643 | 166520 | ±166520 | 3.668 |
| u26 | 522.345 | 15.716 | 552574600 | ±184163236 | 2338673 | ±1692113 | 3.457 |
| u27 | 525.377 | 16.667 | 57351758 | ±10047775 | 83005407 | ±4302844 | 3.300 |
| u28 | 741.615 | 17.039 | 81673618 | ±3892400 | 71014623 | ±7762073 | 3.295 |
| u29 | 521.342 | 15.716 | 102354721 | ±12354208 | 131473980 | ±4407973 | 3.294 |
| u30 | 520.342 | 15.734 | 60152381 | ±17927326 | 91013 | ±54557 | 3.279 |
| u31 | 992.674 | 16.037 | 589012 | ±107517 | 366271 | ±151009 | 3.265 |
| u32 | 521.345 | 15.734 | 43283403 | ±8002466 | 38105205 | ±4382916 | 3.254 |
| u33 | 466.329 | 16.911 | 43487226 | ±5585163 | 26433514 | ±4190684 | 3.221 |
| u34 | 198.185 | 20.979 | 36227930 | ±1576630 | 38652623 | ±1983865 | 3.173 |
| u35 | 706.529 | 18.484 | 13253021 | ±4030477 | 1543 | ±1067 | 3.101 |
| u36 | 376.343 | 17.057 | 150337511 | ±27431229 | 205481956 | ±30539527 | 3.034 |
| u37 | 831.571 | 18.306 | 99000568 | ±16577274 | 110430567 | ±38534624 | 2.994 |
| u38 | 782.572 | 18.592 | 2629 | ±1662 | 3024 | ±2060 | 2.940 |
| u39 | 756.553 | 18.303 | 3251564845 | ±426667775 | 3407402617 | ±347253465 | 2.739 |
| u40 | 731.540 | 18.257 | 2898562099 | ±303920747 | 3131354598 | ±214901340 | 2.732 |
| u41 | 780.545 | 18.372 | 4551153905 | ±472520609 | 4592235285 | ±440923455 | 2.727 |
| u42 | 756.546 | 18.313 | 5765390629 | ±526598571 | 5484192639 | ±587969813 | 2.718 |
| u43 | 730.592 | 18.512 | 3657359361 | ±261089769 | 2721877291 | ±507568593 | 2.718 |
| u44 | 619.530 | 20.210 | 920764063 | ±118957152 | 1079237047 | ±63508891 | 2.614 |
| u45 | 773.591 | 18.844 | 445662002 | ±133641197 | 99657 | ±68938 | 2.606 |
| u46 | 704.569 | 18.486 | 66197872 | ±3959390 | 76073281 | ±4180492 | 2.606 |
| u47 | 684.203 | 18.425 | 31797598 | ±8831959 | 4862954 | ±1741034 | 2.605 |
| u48 | 676.546 | 18.084 | 172699 | ±76764 | 275378 | ±114888 | 2.602 |
| u49 | 654.464 | 18.065 | 263318178 | ±43479888 | 553031340 | ±41921877 | 2.582 |
| u50 | 693.563 | 19.663 | 260148577 | ±36121148 | 347327727 | ±21218819 | 2.544 |
| u51 | 823.567 | 17.617 | 456162471 | ±28003191 | 485351227 | ±41532022 | 2.490 |
| u52 | 705.584 | 18.475 | 316456820 | ±18971065 | 313759127 | ±21061369 | 2.462 |
| u53 | 683.545 | 16.502 | 128034542 | ±38443628 | 2224954 | ±1478001 | 2.425 |
| u54 | 760.588 | 18.980 | 6981175 | ±1779037 | 13674709 | ±3951137 | 2.414 |
| u55 | 619.606 | 16.989 | 40417003 | ±2539672 | 40187869 | ±2686797 | 2.399 |
| u56 | 679.509 | 18.085 | 16592808 | ±4864765 | 471314 | ±227073 | 2.365 |
| u57 | 169.058 | 0.632 | 46031037 | ±2973923 | 46134343 | ±3130061 | 2.365 |
| u58 | 311.315 | 17.000 | 18514234 | ±5522270 | 206884 | ±54016 | 2.333 |
| u59 | 810.580 | 18.739 | 26925 | ±8846 | 31271 | ±15255 | 2.323 |
| u60 | 525.374 | 16.669 | 326799108 | ±24451179 | 363795981 | ±28653859 | 2.240 |
| u61 | 828.595 | 19.539 | 171694888 | ±52108980 | 2595 | ±2595 | 2.236 |
| u62 | 774.660 | 19.712 | 8516403 | ±1434889 | 13887677 | ±683946 | 2.225 |
| u63 | 797.587 | 18.821 | 32998190 | ±4081701 | 33108666 | ±1467033 | 2.224 |
| u64 | 242.925 | 0.604 | 38486785 | ±3252840 | 30001225 | ±2365604 | 2.216 |
| u65 | 721.598 | 20.292 | 983699458 | ±108027667 | 1227689704 | ±38181997 | 2.198 |
| u66 | 521.419 | 16.859 | 1767793026 | ±89292644 | 1888788110 | ±75393614 | 2.180 |
| u67 | 360.320 | 17.057 | 1100006795 | ±89547446 | 909116062 | ±158387460 | 2.168 |
| u68 | 991.677 | 16.042 | 835345014 | ±250176495 | 5775567 | ±3129161 | 2.139 |
| u69 | 643.526 | 19.818 | 25811547 | ±1127432 | 29054831 | ±1957611 | 2.111 |
| u70 | 993.676 | 16.037 | 10459095 | ±2935685 | 1116372 | ±1060732 | 2.094 |
| u71 | 809.654 | 20.003 | 171026094 | ±22570156 | 247326155 | ±13146459 | 2.086 |
| u72 | 723.531 | 18.150 | 80567800 | ±24718373 | 112341 | ±54400 | 2.068 |
| u73 | 522.348 | 15.734 | 614945758 | ±82145697 | 765178503 | ±61723197 | 2.036 |
| u74 | 617.341 | 15.944 | 313728921 | ±92438198 | 36987259 | ±11378890 | 2.028 |
| u75 | 830.573 | 18.288 | 164338538 | ±14927941 | 164441924 | ±17292799 | 1.992 |
| u76 | 296.259 | 14.218 | 60098845 | ±19145675 | 1573515 | ±542172 | 1.990 |
| u77 | 619.616 | 17.000 | 32165642 | ±4488533 | 40750365 | ±3399874 | 1.965 |
| u78 | 532.340 | 16.386 | 18380337 | ±4742201 | 3901463 | ±777506 | 1.949 |
| u79 | 546.345 | 16.691 | 23525 | ±13132 | 11400 | ±8249 | 1.911 |
| u80 | 557.445 | 17.037 | 263176774 | ±32294014 | 344567934 | ±11225547 | 1.892 |
| u81 | 612.181 | 18.150 | 1423003289 | ±199302995 | 1944516875 | ±50622085 | 1.888 |
| u82 | 256.264 | 16.274 | 1995018993 | ±49128490 | 2023922054 | ±52102821 | 1.869 |
| u83 | 254.247 | 15.713 | 2125142962 | ±44038416 | 2242548942 | ±67171856 | 1.860 |
| u84 | 254.249 | 15.699 | 341506687 | ±12945078 | 304719309 | ±29250218 | 1.859 |
| u85 | 636.556 | 19.344 | 1622962023 | ±150975478 | 1247675861 | ±234925704 | 1.856 |
| u86 | 785.587 | 18.773 | 325041190 | ±15088698 | 319566754 | ±26768168 | 1.850 |
| u87 | 523.361 | 16.207 | 101808027 | ±12705698 | 105518810 | ±12419998 | 1.825 |
| u88 | 306.278 | 16.893 | 36340757 | ±11857396 | 110491 | ±32767 | 1.811 |
| u89 | 698.527 | 18.085 | 88329045 | ±10559245 | 120091347 | ±3617065 | 1.805 |
| u90 | 550.513 | 19.253 | 90451305 | ±7693990 | 73809824 | ±10995783 | 1.798 |
| u91 | 647.458 | 19.896 | 129744312 | ±11490519 | 140011189 | ±12801658 | 1.787 |
| u92 | 642.514 | 19.338 | 48815685 | ±15015562 | 9048906 | ±8824851 | 1.783 |
| u93 | 361.321 | 17.050 | 885115611 | ±120687811 | 1072039379 | ±68544605 | 1.765 |
| u94 | 358.303 | 16.646 | 445855900 | ±133930061 | 1109598 | ±800967 | 1.761 |
| u95 | 586.540 | 19.663 | 33919030 | ±2928018 | 31378748 | ±2776796 | 1.755 |
| u96 | 543.327 | 15.295 | 12277776 | ±3762286 | 127476 | ±52152 | 1.752 |
| u97 | 608.317 | 16.851 | 85721044 | ±3392485 | 81397500 | ±5883784 | 1.749 |
| u98 | 744.557 | 18.437 | 33777873 | ±9913556 | 1056381 | ±386809 | 1.741 |
| u99 | 482.360 | 16.399 | 7433718 | ±1466767 | 12675703 | ±740149 | 1.710 |
| u100 | 563.553 | 16.435 | 52495967 | ±2011906 | 50530677 | ±2832928 | 1.708 |
| u101 | 548.495 | 19.246 | 19895718 | ±6035679 | 280394 | ±75063 | 1.693 |
| u102 | 360.362 | 14.886 | 7761171 | ±1360304 | 11613036 | ±829465 | 1.679 |
| u103 | 568.588 | 16.909 | 45459525 | ±6703031 | 60973968 | ±5346462 | 1.667 |
| u104 | 359.298 | 13.315 | 19274115 | ±5816577 | 60162 | ±32553 | 1.624 |
| u105 | 643.519 | 19.809 | 100210563 | ±13501750 | 132954453 | ±6809120 | 1.623 |
| u106 | 304.257 | 16.434 | 54194835 | ±16359063 | 268150 | ±50810 | 1.618 |
| u107 | 232.928 | 0.553 | 13679265 | ±1127333 | 38122373 | ±17595708 | 1.605 |
| u108 | 456.339 | 16.938 | 71717453 | ±13432296 | 106990517 | ±4844199 | 1.604 |
| u109 | 579.534 | 20.197 | 47550513 | ±13881513 | 2135674 | ±1062813 | 1.591 |
| u110 | 360.318 | 17.050 | 44467182 | ±1751698 | 42707862 | ±3024876 | 1.581 |
| u111 | 480.310 | 16.226 | 69642586 | ±8471738 | 97719188 | ±3743672 | 1.575 |
| u112 | 743.581 | 18.830 | 38040492 | ±11201728 | 913116 | ±163703 | 1.553 |
| u113 | 744.544 | 18.450 | 213026657 | ±32768248 | 190632475 | ±20553511 | 1.550 |
| u114 | 828.554 | 18.538 | 106622836 | ±32360500 | 26016213 | ±11366892 | 1.547 |
| u115 | 198.097 | 0.662 | 691052733 | ±84017884 | 930691181 | ±84608736 | 1.543 |
| u116 | 568.379 | 17.251 | 292396712 | ±91183713 | 1439816 | ±571821 | 1.528 |
| u117 | 821.623 | 19.208 | 37537816 | ±6499561 | 61149937 | ±5674011 | 1.524 |
| u118 | 744.553 | 18.434 | 24342905 | ±5200738 | 8229007 | ±1818697 | 1.501 |
| u119 | 718.573 | 19.153 | 202558736 | ±19122669 | 205186459 | ±21661526 | 1.496 |
| u120 | 701.551 | 18.157 | 75701793 | ±24485296 | 1054108 | ±596532 | 1.490 |
| u121 | 794.609 | 18.907 | 37329469 | ±1422563 | 38778353 | ±1875614 | 1.489 |
| u122 | 104.108 | 16.185 | 30552482 | ±6174306 | 35618266 | ±7431330 | 1.464 |
| u123 | 313.273 | 20.197 | 24258262 | ±4768223 | 18212635 | ±8773042 | 1.462 |
| u124 | 854.612 | 20.059 | 17801012 | ±2904835 | 30127020 | ±4619598 | 1.420 |
| u125 | 832.569 | 19.034 | 12374550 | ±3718087 | 545871 | ±164455 | 1.412 |
| u126 | 523.358 | 16.154 | 106816011 | ±13971765 | 129361310 | ±8774109 | 1.410 |
| u127 | 244.190 | 16.802 | 129398024 | ±4820611 | 96613555 | ±15760732 | 1.410 |
| u128 | 823.643 | 19.389 | 73681027 | ±4706290 | 76784866 | ±3555536 | 1.402 |
| u129 | 902.398 | 16.966 | 5050485 | ±782792 | 4754062 | ±397005 | 1.400 |
| u130 | 728.526 | 18.016 | 55328243 | ±5961063 | 62535028 | ±3711372 | 1.340 |
| u131 | 993.651 | 18.951 | 53042490 | ±4387895 | 41078440 | ±7139302 | 1.317 |
| u132 | 757.560 | 18.314 | 53765318 | ±14465244 | 70479093 | ±14368845 | 1.304 |
| u133 | 757.556 | 18.303 | 37530396 | ±7231957 | 14322892 | ±3091546 | 1.299 |
| u134 | 358.369 | 15.540 | 2823954 | ±1107430 | 4079617 | ±991565 | 1.298 |
| u135 | 509.379 | 16.540 | 24934719 | ±4350107 | 31453033 | ±4255867 | 1.292 |
| u136 | 90.055 | 0.642 | 8460000 | ±3002361 | 13297 | ±7042 | 1.277 |
| u137 | 405.379 | 17.501 | 17421609 | ±3128761 | 31555676 | ±2321195 | 1.271 |
| u138 | 305.135 | 14.042 | 25519479 | ±1915309 | 25449971 | ±3612569 | 1.269 |
| u139 | 838.563 | 17.592 | 68085785 | ±9108581 | 81794719 | ±6021758 | 1.261 |
| u140 | 138.992 | 0.552 | 52524327 | ±10748719 | 23259184 | ±6738077 | 1.247 |
| u141 | 676.683 | 17.458 | 123406048 | ±10485777 | 131055277 | ±8912756 | 1.244 |
| u142 | 797.629 | 19.337 | 53957620 | ±16148453 | 669600 | ±218685 | 1.234 |
| u143 | 362.927 | 0.600 | 379838 | ±103572 | 370391 | ±309936 | 1.233 |
| u144 | 186.221 | 12.975 | 485044977 | ±26900008 | 515909803 | ±22354979 | 1.231 |
| u145 | 731.545 | 18.249 | 232140175 | ±69562549 | 0 | ± | 1.229 |
| u146 | 133.106 | 1.857 | 82626213 | ±6009745 | 68640212 | ±9658057 | 1.226 |
| u147 | 854.573 | 18.219 | 38914653 | ±10471312 | 4537481 | ±1510451 | 1.225 |
| u148 | 275.065 | 11.765 | 56852882 | ±9849461 | 80101381 | ±6847402 | 1.219 |
| u149 | 256.263 | 16.278 | 68146096 | ±6083828 | 53662142 | ±12741362 | 1.218 |
| u150 | 207.159 | 9.337 | 330618188 | ±18675604 | 324720975 | ±29777280 | 1.215 |
| u151 | 814.625 | 19.218 | 43357240 | ±7269405 | 66255965 | ±5386202 | 1.204 |
| u152 | 250.940 | 0.542 | 2738447 | ±2195714 | 2349948 | ±1403921 | 1.203 |
| u153 | 820.610 | 19.262 | 2576492 | ±2206594 | 1123383 | ±1014101 | 1.193 |
| u154 | 198.186 | 20.841 | 17118005 | ±4861521 | 14395901 | ±877933 | 1.193 |
| u155 | 565.567 | 19.392 | 21151874 | ±3458130 | 22927895 | ±1625455 | 1.191 |
| u156 | 677.548 | 18.082 | 11595657 | ±3732839 | 173031 | ±68494 | 1.177 |
| u157 | 403.232 | 14.897 | 44521491 | ±5908846 | 59958137 | ±3034052 | 1.169 |
| u158 | 878.699 | 20.553 | 29099938 | ±8498714 | 761154 | ±253717 | 1.165 |
| u159 | 620.616 | 16.992 | 143670274 | ±19404253 | 209126053 | ±7787805 | 1.164 |
| u160 | 522.355 | 16.154 | 80698261 | ±24418511 | 89890 | ±27216 | 1.149 |
| u161 | 483.329 | 15.643 | 4670596 | ±433420 | 5987149 | ±356577 | 1.140 |
| u162 | 731.596 | 18.927 | 33059783 | ±3417025 | 30353701 | ±3186401 | 1.134 |
| u163 | 532.337 | 16.366 | 10434775 | ±3518126 | 53401 | ±12034 | 1.130 |
| u164 | 829.553 | 18.516 | 487813350 | ±82644750 | 708584541 | ±21376890 | 1.126 |
| u165 | 295.102 | 0.697 | 682983648 | ±29224330 | 594840888 | ±79436289 | 1.124 |
| u166 | 716.643 | 20.292 | 43389847 | ±11258462 | 5347591 | ±1991052 | 1.117 |
| u167 | 314.278 | 16.499 | 35104271 | ±5983077 | 52543867 | ±1523620 | 1.104 |
| u168 | 855.571 | 18.237 | 51705665 | ±1319783 | 47404890 | ±3710334 | 1.104 |
| u169 | 121.072 | 0.681 | 146901244 | ±15360543 | 125300040 | ±28724915 | 1.096 |
| u170 | 111.021 | 19.927 | 41928760 | ±13029739 | 303262 | ±111312 | 1.096 |
| u171 | 95.086 | 17.050 | 664607520 | ±20665308 | 640593151 | ±40725164 | 1.094 |
| u172 | 88.076 | 8.717 | 24684053 | ±1987289 | 27142502 | ±1922798 | 1.057 |
| u173 | 360.325 | 17.458 | 318313768 | ±37825583 | 364986477 | ±33172965 | 1.051 |
| u174 | 537.166 | 17.872 | 129492813 | ±38982098 | 7429058 | ±5153742 | 1.038 |
| u175 | 433.354 | 16.938 | 46574990 | ±5514842 | 52204617 | ±3718755 | 1.034 |
| u176 | 724.526 | 18.789 | 46174243 | ±4096915 | 36005305 | ±6777386 | 1.033 |
| u177 | 760.584 | 18.952 | 28575185 | ±4260852 | 35131866 | ±3261976 | 1.030 |
| u178 | 730.597 | 18.540 | 15826991 | ±3160529 | 7414635 | ±2669608 | 1.026 |
| u179 | 550.387 | 16.758 | 122048764 | ±16781722 | 134815205 | ±22558228 | 1.023 |
| u180 | 823.639 | 19.376 | 34743710 | ±13026953 | 27668 | ±11122 | 1.017 |
| u181 | 537.538 | 18.868 | 25797319 | ±1263803 | 25585524 | ±1265174 | 1.010 |
| u182 | 722.602 | 20.292 | 25522368 | ±6628010 | 21185859 | ±3223100 | 1.005 |
| u183 | 760.640 | 19.393 | 10136895 | ±4556540 | 885975 | ±367265 | 1.002 |
| u184 | 877.690 | 20.043 | 1833075 | ±614111 | 1193530 | ±278611 | 1.002 |

Mean ± SE. u: unknown, VIP: variable important in the projection.

**Supplementary Table S3. AUCs for featured biomarkers.**

|  | **AUC** | **S.E.** | **95% CI** | ***p*** |
| --- | --- | --- | --- | --- |
| BMI (kg/m^2^) | 0.809 | 0.080 | 0.621-0.930 | <0.001 |
| Triglyceride (mg/dL)*^†^* | 0.863 | 0.080 | 0.684-0.961 | <0.001 |
| HDL cholesterol (mg/dL)*^†^* | 0.770 | 0.088 | 0.577-0.905 | 0.002 |
| Gamma-GT (U/L)*^†^* | 0.721 | 0.109 | 0.524-0.870 | 0.043 |
| Leptin (ng/mL)*^†^* | 0.775 | 0.089 | 0.582-0.908 | 0.002 |
| Fat percentage (%)*^†^* | 0.762 | 0.094 | 0.569-0.900 | 0.005 |
| Whole fat area (cm^2^) at L1*^†^* | 0.748 | 0.092 | 0.553-0.889 | 0.007 |
| Subcutaneous fat area (cm^2^) at L1*^†^* | 0.750 | 0.100 | 0.555-0.891 | 0.012 |
| 4-Aminobutyraldehyde | 0.804 | 0.082 | 0.615-0.927 | <0.001 |
| 4'-apo-β-carotenal | 0.750 | 0.094 | 0.555-0.891 | 0.008 |

All features were selected based on the *p*-values derived from the Mann-Whitney *U* test at 12 weeks. *^†^*Tested by logarithmic transformation.
